# Coverage with Selected Vaccines and Exemption from School Vaccine Requirements Among Children in Kindergarten — United States, 2022–23 School Year

**DOI:** 10.15585/mmwr.mm7245a2

**Published:** 2023-11-10

**Authors:** Ranee Seither, Oyindamola Bidemi Yusuf, Devon Dramann, Kayla Calhoun, Agnes Mugerwa-Kasujja, Cynthia L. Knighton

**Affiliations:** ^1^Immunization Services Division, National Center for Immunization and Respiratory Diseases, CDC; ^2^Certified Technical Experts, Inc., Montgomery, Alabama; ^3^Association of Schools and Programs of Public Health, Washington, DC.

**Summary**

**What is already known about this topic?**

From the 2019–20 to the 2021–22 school year, national coverage with state-required vaccines among kindergartners declined from 95% to approximately 93%, ranging from 92.7% for diphtheria, tetanus, and acellular pertussis vaccine (DTaP) to 93.1% for polio.

**What is added by this report?**

During the 2022–23 school year, coverage remained near 93% for all reported vaccines, ranging from 92.7% for DTaP to 93.1% for measles, mumps, and rubella and polio. The exemption rate increased 0.4 percentage points to 3.0%. Exemptions increased in 41 states, exceeding 5% in 10 states.

**What are the implications for public health practice?**

Exemptions >5% limit the level of achievable vaccination coverage, which increases the risk for outbreaks of vaccine-preventable diseases. Vaccination before school entry or during provisional enrollment periods could reduce exemptions resulting from barriers to vaccination during the COVID-19 pandemic.

## Abstract

U.S. states and local jurisdictions set vaccination requirements for school attendance and conditions and procedures for exemptions from these requirements. States annually report data to CDC on the number of children in kindergarten who meet, are exempt from, or are in the process of meeting requirements. National- and state-level estimates for complete vaccination with measles, mumps, and rubella vaccine (MMR); diphtheria, tetanus, and acellular pertussis vaccine (DTaP); poliovirus vaccine (polio); and varicella vaccine (VAR); exemptions from vaccination; and legally allowed kindergarten attendance while meeting requirements were based on data reported by 49 states and the District of Columbia (DC) for the 2022–23 school year. This kindergarten class became age-eligible to complete most state-required vaccinations during the COVID-19 pandemic. National coverage remained near 93% for all vaccines; exemptions were low but increased to 3%, compared with those during the 2021–22 school year (2.6%). At the state level, coverage with MMR, DTaP, polio, and VAR decreased in 29, 31, 28, and 25 states, respectively, compared with coverage during the 2021–22 school year. Exemptions increased in 40 states and DC, with 10 states reporting an exemption from at least one vaccine for >5% of kindergartners. Schools and providers should work to ensure that students are vaccinated before school entry, such as during the enrollment process, which is often several months before school starts. State and local provisional enrollment periods that allow students to attend school while on a catch-up schedule also provide the opportunity to fully vaccinate students and to prevent nonmedical exemptions resulting from lingering undervaccination due to COVID-19 pandemic–related barriers to vaccination, such as reduced access to vaccination appointments.

## Introduction

State and local school vaccination requirements promote vaccination to protect students, schools, and communities against vaccine-preventable diseases (*1*). After 10 years of near 95% nationwide vaccination coverage, measles, mumps, and rubella vaccine (MMR)*; diphtheria, tetanus, and acellular pertussis vaccine (DTaP)^†^; poliovirus vaccine (polio)^§^; and varicella vaccine (VAR)^¶^ coverage declined approximately 1 percentage point during the 2020–21 school year and fell an additional percentage point during the 2021–22 school year, to approximately 93% (*2*). For both the 2020–21 and 2021–22 school years, states reported impacts of the COVID-19 pandemic and response for both vaccine administration and data collection (*3*,*4*). This analysis summarizes data collected and reported by state and local immunization programs** on vaccination coverage and exemptions to vaccination among kindergartners in 49 states^††^ and the District of Columbia (DC), and provisional enrollment or grace period status for kindergartners in 28 states^§§^ for the 2022–23 school year.

## Methods

### Data Collection and Reporting

As mandated by state and local school entry requirements, either parents provide children’s vaccination or exemption documentation to schools, or schools obtain records from state immunization information systems. Federally funded immunization programs work with departments of education, local health departments, school nurses, and other school personnel to assess the vaccination and exemption status of children enrolled in public and private kindergartens and to report unweighted counts, aggregated by school type, to CDC via a questionnaire in the Secure Access Management System, a federal, web-based platform that provides authorized personnel with secure access to public health applications operated by CDC. CDC uses these data to produce state- and national-level estimates of vaccination coverage among children in kindergarten. During the 2022–23 school year, 49 states and DC reported coverage with all state-required vaccines and exemption data for public school kindergartners; 48 states and DC reported coverage with all state-required vaccines and exemption data for private school kindergartners.^¶¶^ Data from cities were included with their state data. State-level, national, and median coverage with the state-required number of DTaP, MMR, polio, and VAR doses are reported. Hepatitis B vaccination coverage is not included in this report but is available at SchoolVaxView (*2*). Twenty-eight states reported the number of kindergartners who were attending school under a grace period (attendance without proof of complete vaccination or exemption during a set number of days) or provisional enrollment (school attendance while completing a catch-up vaccination schedule). All counts were current as of the time of the assessment by the state immunization program.***

### Data Analyses

National estimates, medians, and summary measures include only U.S. states and DC. Vaccination coverage and exemption estimates were adjusted on the basis of survey type and response rate.^†††^ National estimates measure coverage and exemptions among all kindergartners, whereas medians indicate the midpoint of state-level coverage, irrespective of population size. During the 2022–23 school year, immunization programs reported 3,832,381 children enrolled in kindergarten in 49 states and DC.^§§§^ Reported estimates are based on 3,559,366 (92.9%) children who were surveyed for vaccination coverage, 3,711,948 (96.9%) surveyed for exemptions, and 2,683,880 (70.0%) surveyed for grace period and provisional enrollment status. Potentially achievable coverage with MMR (the sum of the percentage of children who were up to date with 2 doses of MMR and those not up to date but nonexempt) was calculated for each state. Nonexempt students (those who do not have medical or nonmedical exemptions and who are not up to date) include those who were provisionally enrolled in kindergarten, in a grace period, or otherwise without documentation of complete vaccination. Vaccination assessments varied by state because of differences in required vaccines and required numbers of doses, vaccines assessed, methods of data collection, and data reported (Supplementary Table 1, https://stacks.cdc.gov/view/cdc/134738). Kindergartners were considered up to date with a given vaccine if they received all doses for that vaccine required for school entry, except in nine states^¶¶¶^ that reported kindergartners as up to date for any vaccine only if they had received all doses of all vaccines required for school entry. All but four states reported the number of kindergartners with an exemption for least one vaccine.**** SAS software (version 9.4; SAS Institute) was used for all analyses. This activity was reviewed by CDC, deemed not research, and was conducted consistent with applicable federal law and CDC policy.^††††^

## Results

### Vaccination Coverage

Nationally, 2-dose MMR coverage was 93.1% (range = 81.3% [Idaho] to ≥98.4% [Mississippi]), with coverage of ≥95% reported by 13 states and <90% by 12 states and DC (Table). DTaP coverage was 92.7% (range = 81.0% [Idaho] to ≥98.4% [Mississippi]); ≥95% coverage was reported by 11 states and <90% by 14 states and DC. Polio coverage was 93.1% (range = 81.8% [Idaho] to ≥98.4% [Mississippi]), with ≥95% coverage reported by 13 states and <90% by 12 states and DC. VAR coverage was 92.9% (range = 80.7% [Idaho] to ≥98.4% [Mississippi]), with 11 states reporting ≥95% coverage and 12 states and DC reporting <90% coverage. Coverage during the 2022–23 school year decreased in most states for all vaccines compared with the 2021–22 school year. (Supplementary Figure, https://stacks.cdc.gov/view/cdc/134740).

**TABLE:** Estimated* coverage^†^ with measles, mumps, and rubella; diphtheria, tetanus, and acellular pertussis; poliovirus; and varicella vaccines; grace period or provisional enrollment^§^; and any exemption^¶^^,^** among kindergartners, by immunization program — United States,^††^ 2022–23 school year

Immunization program	Kindergarten population^§§^	Percentage							PP change in any exemption from 2021–22 school year
		Surveyed^¶¶^	2 doses of MMR***	5 doses of DTaP^†††^	4 doses of polio^§§§^	2 doses of VAR^¶¶¶^	Grace period or provisional enrollment	Any exemption	
National estimate****	**3,832,381**	**92.9**	**93.1**	**92.7**	**93.1**	**92.9**	**2.5**	**3.0**	**0.4**
Median****	**—**	**—**	**92.1**	**91.9**	**92.2**	**92.7**	**2.0**	**3.3**	**0.6**
**U.S. state/Jurisdiction**									
Alabama^††††,§§§§^	59,113	100.0	≥93.9	≥93.9	≥93.9	≥93.9	NP	2.0	0.3
Alaska^§§§§,¶¶¶¶^	9,650	88.8	83.6	83.8	84.4	81.8	NR	5.7	1.1
Arizona*****	80,814	97.7	89.9	89.6	90.3	94.1	NR	7.4	0.6
Arkansas	38,358	95.8	91.9	90.6	90.7	91.1	9.2	3.1	0.6
California^§§§§,^*****^,†††††^	541,132	>99.9	96.5	95.6	96.3	96.1	1.5	0.2	–0.1
Colorado	65,576	97.2	87.0	87.2	87.0	85.9	≥0.6	≥4.3	1.1
Connecticut^††††,§§§§^	35,580	100.0	97.3	97.3	97.3	97.0	NP	0.8	–1.5
Delaware^§§§§,†††††^	10,674	9.7	95.1	93.8	94.0	94.0	NR	2.1	0.9
District of Columbia^††††,§§§§^	8,064	100.0	87.5	85.0	87.8	86.8	NR	1.3	0.8
Florida^§§§§^	230,309	97.7	≥90.6	≥90.6	≥90.6	≥90.6	4.7	4.5	0.6
Georgia^††††,§§§§^	123,771	100.0	≥88.1	≥88.1	≥88.1	≥88.1	0.5	3.8	–0.9
Hawaii^§§§§^	13,195	8.1	86.4	87.0	87.0	84.4	0.5	6.4	3.0
Idaho	23,721	99.3	81.3	81.0	81.8	80.7	1.9	12.1	2.3
Illinois^††††,§§§§^	135,332	100.0	91.7	91.5	91.4	91.3	NR	≥2.1	0.4
Indiana^§§§§,§§§§§^	81,307	87.5	92.0	83.0	88.8	91.6	NR	2.8	0.4
Iowa^††††,§§§§^	39,178	100.0	≥89.9	≥89.9	≥89.9	≥89.9	5.3	3.0	0.6
Kansas^§§§§,†††††,§§§§§,¶¶¶¶¶^	35,543	30.8	91.6	90.5	92.2	90.8	NP	2.9	0.6
Kentucky^§§§§,†††††,§§§§§^	54,742	96.9	≥90.1	≥90.6	≥91.2	≥89.8	NR	1.7	0.4
Louisiana^††††^	54,314	100.0	92.2	93.1	98.3	93.6	NP	2.3	1.2
Maine	12,403	93.9	96.8	96.6	96.8	96.6	NR	0.9	–0.9
Maryland^††††,§§§§,†††††^	59,684	100.0	96.7	96.9	97.2	96.6	NR	1.9	0.4
Massachusetts^††††,§§§§,†††††^	66,041	100.0	96.5	96.2	96.3	96.0	NP	1.4	0.4
Michigan^††††^	113,678	100.0	92.9	93.1	93.7	92.9	1.0	5.4	0.9
Minnesota	68,152	97.9	87.6	88.3	88.6	87.9	NR	≥4.5	0.8
Mississippi^††††,§§§§,^*****	36,048	100.0	≥98.4	≥98.4	≥98.4	≥98.4	1.0	0.2	0.1
Missouri^††††,§§§§^	69,126	100.0	91.3	91.1	91.5	90.8	NR	≥3.8	0.8
Montana	NR	NR	NR	NR	NR	NR	NR	NR	NA
Nebraska^††††,§§§§,†††††^	23,176	100.0	95.1	95.7	97.0	94.9	2.6	2.6	0.1
Nevada^§§§§^	34,333	89.1	92.8	92.2	92.8	92.6	1.7	5.6	0.8
New Hampshire ^††††,§§§§,§§§§§^	11,332	100.0	≥89.4	≥89.4	≥89.4	≥89.4	4.5	3.4	0
New Jersey^††††,§§§§,§§§§§^	104,468	100.0	≥94.3	≥94.3	≥94.3	≥94.3	1.1	3.2	0.6
New Mexico^††††,§§§§^	21,068	100.0	94.9	94.7	95.0	94.4	2.0	1.5	0.1
New York (including NYC) ^§§§§,^*****	205,906	96.6	97.9	97.2	97.5	97.5	2.3	0.1	0
NYC^§§§§,^*****	85,379	97.6	97.3	96.3	96.6	96.7	2.3	0.1	0
North Carolina ^§§§§,†††††,§§§§§^	125,679	83.1	93.8	93.7	93.9	93.6	1.6	2.4	0.5
North Dakota	10,554	99.4	92.0	91.8	91.9	91.4	NR	5.1	–0.2
Ohio	134,893	93.7	89.3	89.4	89.7	88.8	5.9	3.8	0.8
Oklahoma^†††††^	52,548	89.5	89.6	90.0	91.0	94.6	NR	4.7	1.2
Oregon^††††,†††††^	40,963	100.0	91.9	90.9	91.5	94.1	NR	8.2	1.2
Pennsylvania	137,259	97.2	94.0	94.3	94.1	93.7	2.3	3.8	0.5
Rhode Island^§§§§,†††††,§§§§§^	10,532	96.5	96.9	96.9	96.9	96.3	0.9	1.5	0.3
South Carolina^§§§§,¶¶¶¶¶^	58,878	28.1	93.2	92.1	92.4	92.8	4.7	4.1	0.7
South Dakota^††††,§§§§^	12,081	100.0	92.5	92.2	92.3	92.0	NR	4.1	0.6
Tennessee^††††,§§§§,§§§§§^	79,692	100.0	95.4	94.8	95.0	95.1	2.0	3.2	0.8
Texas (including Houston)^†††††,§§§§§^	381,680	98.0	94.2	93.8	94.1	93.7	1.9	3.5	0.6
Houston^†††††,§§§§§^	37,664	98.8	91.3	90.7	91.0	90.6	2.6	2.3	0.8
Utah^††††,^******	46,635	100.0	90.0	89.7	89.9	89.6	3.7	8.1	NA
Vermont^††††,§§§§^	5,816	100.0	93.1	92.8	92.8	92.6	6.3	3.6	0.3
Virginia^§§§§,¶¶¶¶¶^	93,271	1.6	95.8	97.8	94.2	95.6	NR	2.2	0.4
Washington^§§§§§^	86,284	97.9	91.4	90.1	90.2	90.1	1.6	4.0	0.3
West Virginia^§§§§,^*****^,§§§§§,†††††^	19,175	86.1	≥95.6	≥95.6	≥95.6	≥95.6	NR	<0.1	0
Wisconsin^†††††^	63,593	93.9	86.5	87.0	88.2	85.9	5.7	7.2	0.9
Wyoming^††††,§§§§^	7,060	100.0	90.8	89.4	90.1	90.5	2.4	4.8	0.9
**Territories and freely associated states**									
American Samoa^††††^	NR	NR	NR	NR	NR	NR	NR	NR	NA
Federated States of Micronesia^††††^	1,595	100.0	92.2	77.6	82.7	NReq	NR	NR	NA
Guam^††††,§§§§^	2,079	100.0	91.0	86.0	89.1	NReq	NR	NR	NA
Marshall Islands^††††, §§§§,^*^****^	860	100.0	98.1	89.2	90.3	NReq	NR	NR	NA
Northern Mariana Islands^††††^	791	100.0	93.4	98.0	97.5	91.8	NR	0	0
Palau^††††^	261	100.0	≥81.2	≥81.2	≥81.2	NReq	NR	0	NA
Puerto Rico^§§§§^	21,255	9.3	92.8	95.2	96.7	92.9	NR	1.1	–0.7
U.S. Virgin Islands	NR	NR	NR	NR	NR	NR	NR	NR	NA

**Abbreviations:** DTaP = diphtheria, tetanus, and acellular pertussis vaccine; DTP = diphtheria and tetanus toxoids and pertussis vaccine; MMR = measles, mumps, and rubella vaccine; polio = poliovirus vaccine; NA = not available; NP = no grace period or provisional policy; NR = not reported to CDC; NReq = not required; NYC = New York City; PP = percentage point; VAR = varicella vaccine.

* Estimates adjusted for nonresponse and weighted for sampling where appropriate.

^†^ Estimates based on a completed vaccination series (i.e., not vaccine-specific) use the “≥” symbol. Coverage might include history of disease or laboratory evidence of immunity. In Kentucky, public schools reported numbers of children up to date with specific vaccines, and most private schools reported numbers of children who received all doses of all vaccines required for school entry.

^§^ A grace period is a set number of days during which a student can be enrolled and attend school without proof of complete vaccination or exemption. Provisional enrollment allows a student without complete vaccination or exemption to attend school while completing a catch-up vaccination schedule. In states with one or both of these policies, the estimates represent the number of kindergartners who were within a grace period, were provisionally enrolled, or were in a combination of these categories.

^¶^ Some programs did not report the number of children with exemptions, but instead reported the number of exemptions for each vaccine, which could count some children more than once. Lower bounds of the percentage of children with any exemptions were estimated using the individual vaccines with the highest number of exemptions. Estimates based on vaccine-specific exemptions use the “≥” symbol.

** Exemptions, grace period or provisional enrollment, and vaccine coverage status might not be mutually exclusive. Some children enrolled under a grace period or provisional enrollment might be exempt from one or more vaccinations, and children with exemptions might be fully vaccinated with one or more required vaccines.

^††^ Includes five territories and three freely associated states.

^§§^ The kindergarten population is an approximation provided by each program.

^¶¶^ The number surveyed represents the number surveyed for coverage. Exemption estimates are based on 30,224 kindergartners for Kansas, 58,878 for South Carolina, and 92,424 for Virginia.

*** Most states require 2 doses of MMR; Alaska, New Jersey, and Oregon require 2 doses of measles, 1 dose of mumps, and 1 dose of rubella vaccines. Georgia, New York, New York City, North Carolina, and Virginia require 2 doses of measles and mumps vaccines and 1 dose of rubella vaccine. Iowa requires 2 doses of measles vaccine and 2 doses of rubella vaccine. Wyoming requires 1 dose of MMR for kindergarten entry, allowing students until the day before their seventh birthday to receive their second dose, but reported kindergarten coverage with 2 doses of MMR at the time of the assessment.

^†††^ Pertussis vaccination coverage might include some DTP doses if administered in another country or by a vaccination provider who continued to use DTP after 2000. Most states require 5 doses of DTaP for school entry, or 4 doses if the fourth dose was received on or after the fourth birthday; Maryland and Wisconsin require 4 doses; Nebraska requires 3 doses. The reported coverage estimates represent the percentage of kindergartners with the state-required number of DTaP doses, except for Kentucky, which requires ≥5 but reports ≥4 doses of DTaP. Wyoming requires 4 doses of DTaP for kindergarten entry, allowing students until the day before their seventh birthday to receive their fifth dose, but reported kindergarten coverage with 5 doses of DTaP at the time of the assessment.

^§§§^ Most states require 4 doses of polio vaccine for school entry, or 3 doses if the fourth dose was received on or after the fourth birthday; Maryland and Nebraska require 3 doses. The reported coverage estimates represent the percentage of kindergartners with the state-required number of polio doses, except for Kentucky, which requires ≥4 but reports ≥3 doses of polio. Wyoming requires 3 doses of polio for kindergarten entry, allowing students until the day before their seventh birthday to receive their fourth dose, but reported kindergarten coverage with 4 doses of polio at the time of the assessment.

^¶¶¶^ Most states require 2 doses of VAR for school entry; Alabama, Arizona, New Jersey, Oklahoma, and Oregon require 1 dose. Reporting of VAR status for kindergartners with a history of varicella disease varied within and among states; some kindergartners were reported as vaccinated against varicella and others as medically exempt. Wyoming requires 1 dose of VAR for kindergarten entry, allowing students until the day before their seventh birthday to receive their second dose, but reported kindergarten coverage with 2 doses of VAR at the time of the assessment.

**** National coverage and exemption estimates and medians were calculated using data from 49 states and the District of Columbia (i.e., did not include American Samoa, Federated States of Micronesia, Guam, Houston, Marshall Islands, Montana, Northern Mariana Islands, NYC, Palau, Puerto Rico, and the U.S. Virgin Islands). National grace period or provisional enrollment estimates and medians were calculated using data from the 28 states that have either a grace period or provisional enrollment policy and reported relevant data to CDC. Data reported from 3,559,366 kindergartners were assessed for coverage, 3,711,948 for exemptions, and 2,683,880 for grace period or provisional enrollment. Estimates represent rates for populations of coverage and exemptions (3,832,381), and grace period or provisional enrollment (2,763,250).

^††††^ The proportion surveyed is reported as 100% but might be <100% if based on incomplete information about the actual current enrollment.

^§§§§^ Philosophical exemptions were not allowed.

^¶¶¶¶^ Reported public school data only.

***** Religious exemptions were not allowed.

^†††††^ Counted some or all vaccine doses received regardless of Advisory Committee on Immunization Practices–recommended age and time interval; vaccination coverage rates reported might be higher than those for valid doses.

^§§§§§^ Did not include certain types of schools, such as kindergartens in child care facilities, online schools, correctional facilities, or those located on military bases or tribal lands.

^¶¶¶¶¶^ Vaccination coverage data were collected from a sample of kindergartners; exemption data were collected from a census of kindergartners.

****** Utah changed the way data were reported between the 2021–22 and 2022–23 school years and is excluded from this analysis.

### Vaccination Exemptions, Grace Period, and Provisional Enrollment

**FIGURE 1:**
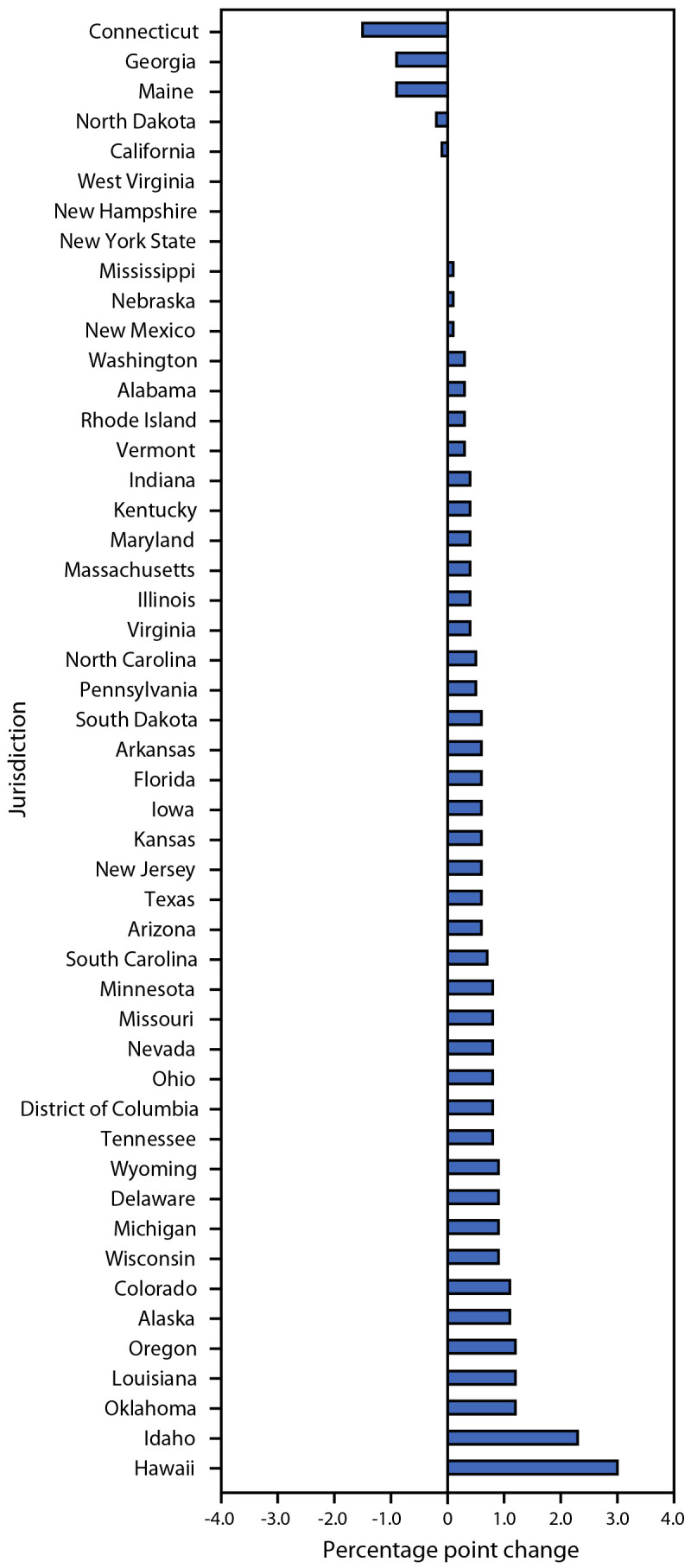
Change in percentage* of kindergartners exempt from one or more vaccinations, by jurisdiction — United States, 2021–22 and 2022–23 school years * Montana did not report kindergarten vaccination coverage for the 2021–22 and 2022–23 school years and is excluded from this analysis. Utah changed the way data were reported between the 2021–22 and 2022–23 school years and is excluded from this analysis.

**FIGURE 2:**
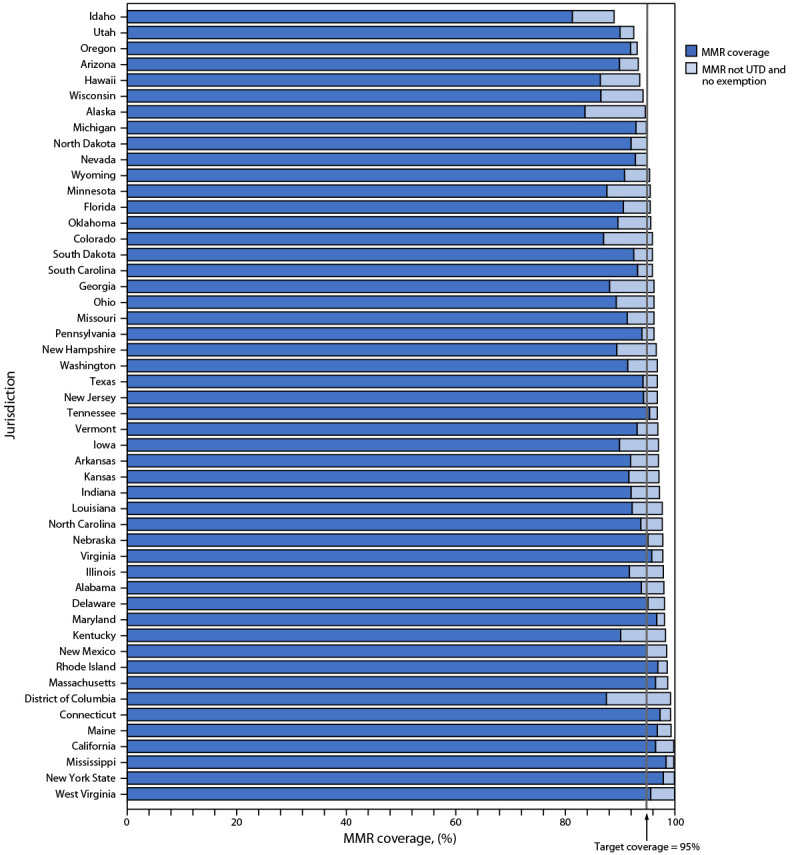
Potentially achievable coverage*^,^^†^^,^^§^ with measles, mumps, and rubella vaccine among kindergartners, by jurisdiction — United States, 2022–23 school year **Abbreviations**: MMR = measles, mumps, and rubella vaccine; UTD = up to date. * Jurisdictions are ranked from lowest to highest potentially achievable coverage. Potentially achievable coverage is estimated as the sum of the percentage of students with UTD MMR and the percentage of students without UTD MMR and without a documented vaccine exemption. Montana did not report kindergarten vaccination coverage for the 2021–22 and 2022–23 school years and is excluded from this analysis. ^†^ The exemptions used to calculate the potential increase in MMR coverage for Alaska, Arizona, Arkansas, Colorado, Delaware, District of Columbia, Idaho, Illinois, Maine, Massachusetts, Michigan, Minnesota, Missouri, Nebraska, Nevada, New York, North Carolina, Oklahoma, Oregon, Rhode Island, Texas, Utah, Vermont, Washington, Wisconsin, and Wyoming are the number of children with exemptions specifically for MMR. For all other jurisdictions, numbers are based on an exemption for any vaccine. **^§^** Potentially achievable coverage in Alaska, Arizona, Hawaii, Idaho, Michigan, Nevada, North Dakota, Oregon, Utah, and Wisconsin is <95%.

Overall, 3.0% of kindergartners had an exemption (0.2% medical and 2.8% nonmedical^§§§§^) from one or more required vaccines (not limited to MMR, DTaP, polio, and VAR) during 2022–23 (range = <0.1% [West Virginia] to 12.1% [Idaho]), compared with 2.6% reported during the 2021–22 school year (Supplementary Table 2, https://stacks.cdc.gov/view/cdc/134739). Exemptions from receipt of one or more vaccines increased in 40 states and DC and increased by at least 1 percentage point in seven states (Figure 1). Nonmedical exemptions account for >90% of reported exemptions, and approximately 100% of the increase in the national exemption rate. Provisional enrollment or grace period attendance in kindergarten was 2.5% among 28 states reporting these data (range = 0.5% [Georgia and Hawaii] to 9.2% [Arkansas]). Nationwide, 3.9% of kindergarten students were not fully vaccinated with MMR and nonexempt. Among the 36 states and DC with MMR coverage <95% during the 2022–23 school year, 10 states reported that >5% of kindergartners were exempt. All but these 10 states could potentially achieve ≥95% MMR coverage if all nonexempt, not up-to-date children were vaccinated, compared with all but four states during the 2021–22 school year (Figure 2).

## Discussion

During the 2022–23 school year, nationwide vaccination coverage among kindergarten children remained approximately 93% for MMR, DTaP, polio, and VAR, similar to that in the 2021–22 school year, lower than the 94% coverage in the 2020–21 school year, and lower still than the 95% coverage during the 2019–20 school year, when children were vaccinated before the COVID-19 public health emergency (*2*–*4*). National MMR coverage among kindergarten students remained below the Healthy People 2030 target of 95% (*5*) for the third consecutive year. Coverage with all four vaccines declined in a majority of states. To address pandemic-related declines in routine immunization coverage across the lifespan, CDC launched the Let’s RISE^¶¶¶¶^ initiative earlier in 2023 and is providing a broad range of communication and enhanced technical assistance, including back-to-school campaigns, to jurisdictions to get routine vaccination coverage back to prepandemic levels as quickly and equitably as possible.

The overall percentage of children with an exemption increased from 2.6% during the 2021–22 school year to 3.0% during the 2022–23 school year, the highest exemption rate ever reported in the United States (*2*). The percentage of children with an exemption increased in 40 states and DC. To achieve the Healthy People 2030 target of 95% MMR coverage, exemptions cannot exceed 5%. State-level exemption rates in excess of 5% prevent 10 states from potentially achieving ≥95% MMR coverage even if all nonexempt kindergartners in 2022–23 were vaccinated, up from four states in 2021–22. National MMR coverage of 93.1% during the 2022–23 school year translates to approximately 250,000 kindergartners who are at risk for measles infection.

### Limitations

The findings in this report are subject to at least four limitations. First, comparisons among states are limited because of variation in state requirements: which vaccines are required, the number of doses required, the date required, the type of documentation accepted, data collection methods, allowable exemptions, definitions of grace period, and provisional enrollment. Second, representativeness might be negatively affected by data collection methods that assess vaccination status at different times, or miss some schools or students (e.g., homeschooled students). Third, vaccination coverage, exemption rates, grace period, or provisional enrollment might be under- or overestimated because of inaccurate or absent documentation. Finally, national coverage estimates for the 2022–23 school year include only 49 of 50 states and DC, and nine states use lower bound estimates; exemption estimates include 49 states and DC, and five states use lower bound estimates.

### Implications for Public Health Practice

Nationwide vaccination coverage among kindergarten students remains below prepandemic levels, and exemptions have increased. Because clusters of undervaccinated children can lead to outbreaks (*6*–*8*), it is important for immunization programs, schools, and providers to make sure children are fully vaccinated before school entry, or before provisional enrollment periods expire. In previous years, nearly all states had the potential to achieve ≥95% coverage if all nonexempt students were vaccinated, but increases in state-level exemptions have reduced that number by 17%, from 48 in 2020–21 to 40 in 2022–23. Exemptions in excess of 5% limit the level of vaccination coverage that can be achieved, which increases the risk of outbreaks of vaccine-preventable diseases. It is not clear whether this reflects a true increase in opposition to vaccination, or if parents are opting for nonmedical exemptions because of barriers to vaccination or out of convenience. Whether because of an increase in hesitancy or barriers to vaccination, the COVID-19 pandemic affected childhood routine vaccination (*9*). Enforcement of school vaccination requirements, school-based vaccination clinics, reminder and recall systems, and follow-up with undervaccinated students have already been shown to be effective in increasing vaccination coverage (*10*). A better understanding of the reasons behind nonmedical exemptions increasing in 40 states and DC, and their impact, could help develop policies that would complement those interventions, to bring higher vaccination coverage and protection against vaccine-preventable diseases within reach of more states.

## Footnotes

* All states except Wyoming require 2 doses of a measles-containing vaccine. Seven states (Alaska, Georgia, New Jersey, New York, North Carolina, Oregon, and Virginia) require only 1 dose of rubella vaccine. Alaska, New Jersey, and Oregon require only 1 dose of mumps vaccine; mumps vaccine is not required in Iowa. Wyoming requires 1 dose of MMR for kindergarten entry, allowing students until the day before their seventh birthday to receive their second dose, but reported kindergarten coverage with 2 doses of MMR at the time of the assessment.

† Nebraska requires 3 doses of DTaP; Maryland and Wisconsin require 4 doses; Wyoming requires 4 doses of DTaP for kindergarten entry, allowing students until the day before their seventh birthday to receive their fifth dose; all other states require 5 doses, unless dose 4 was administered on or after the fourth birthday. The reported coverage estimates represent the percentage of kindergartners with the state-required number of DTaP doses, except for Kentucky, which requires 5 doses of DTaP by age 5 years, but reported 4-dose coverage for kindergartners, and Wyoming, which reported kindergarten coverage with 5 doses of DTaP at the time of the assessment.

§ Two states (Maryland and Nebraska) require only 3 doses of polio; Wyoming requires 3 doses of polio for kindergarten entry, allowing students until the day before their seventh birthday to receive their fourth dose; all other states require 4 doses unless the last dose was given on or after the fourth birthday. The reported coverage estimates represent the percentage of kindergartners with the state-required number of polio doses, except for Kentucky, which requires ≥4 but reports ≥3 doses of polio, and Wyoming, which reported kindergarten coverage with 4 doses of polio at the time of the assessment.

¶ Five states require 1 dose of VAR; 44 states and DC require 2 doses. Wyoming requires 1 dose of VAR for kindergarten entry, allowing students until the day before their seventh birthday to receive their second dose, but reported kindergarten coverage with 2 doses of VAR at the time of the assessment.

** Federally funded immunization programs are in 50 states and DC, five cities, and eight U.S. territories and freely associated states. Two cities (Houston and New York City) reported data to CDC, which were also included in data submitted by their state. State-level data were used to calculate national estimates and medians. Immunization programs in U.S. territories reported vaccination coverage and exemptions; however, these data were not included in national calculations.

†† Montana did not report school vaccination data. Utah changed the way data were reported between the 2021–22 and 2022–23 school years and is excluded from year-to-year comparisons.

§§ Arkansas, California, Colorado, Florida, Georgia, Hawaii, Idaho, Iowa, Michigan, Mississippi, Nebraska, Nevada, New Hampshire, New Jersey, New Mexico, New York, North Carolina, Ohio, Pennsylvania, Rhode Island, South Carolina, Tennessee, Texas, Utah, Vermont, Washington, Wisconsin, and Wyoming reported data on the number of students within a grace period or provisionally enrolled at the time of assessment.

¶¶ Twelve states reported coverage and exemption data for at least some homeschooled kindergartners, either separately, or included with data from public or private schools.

*** Assessment date varied by state and area. Three states assessed schools on the first day of school; nine states assessed schools by December 31; 17 states and DC assessed schools by some other date, ranging from October 1, 2022, to May 15, 2023; and 20 states assessed schools on a rolling basis.

††† Immunization programs that used census or voluntary response provided CDC with data aggregated at the state or local (city or territory) level. Estimates based on these data were adjusted for nonresponse using the inverse of the response rate, stratified by school type (public, private, and homeschool, where available). Programs that used complex sample surveys provided CDC with data aggregated at the school or county level for weighted analyses. Weights were calculated to account for sample design and adjusted for nonresponse.

§§§ These totals are the sums of the kindergartners surveyed among programs reporting data for coverage, exemptions, grace periods, and provisional enrollment. Data from cities and territories were not included in these totals.

¶¶¶ Alabama, Florida, Georgia, Iowa, Mississippi, New Hampshire, New Jersey, and West Virginia considered kindergartners up to date only if they had received all doses of all vaccines required for school entry. In Kentucky, public schools reported numbers of children up to date with specific vaccines, and most private schools reported numbers of children who received all doses of all vaccines required for school entry.

**** Colorado, Illinois, Minnesota, and Missouri did not report the number of kindergartners with an exemption but instead reported the number of exemptions for each vaccine, which could have counted some children more than once. For these states, the percentage of kindergartners exempt from the vaccine with the highest number of exemptions by exemption type (the lower bound of the potential range of exemptions) was included in the national and median exemption rates.

†††† 45 C.F.R. part 46.102(l)(2), 21 C.F.R. part 56; 42 U.S.C. Sect. 241(d); 5 U.S.C. Sect. 552a; 44 U.S.C. Sect. 3501 et seq.

§§§§ Washington was unable to deduplicate data for students with both religious and philosophical exemptions; therefore, the nonmedical exemption type with the highest number of kindergartners (the lower bound of the potential range of nonmedical exemptions) was included in the national and median exemption rates for nonmedical exemptions.

¶¶¶¶
https://www.cdc.gov/vaccines/partners/routine-immunizations-lets-rise.html
